# Green sample preparation for anthocyanin extraction from purple corn: analytical evaluation of pressurized liquid and ultrasound-assisted extraction using sustainable solvents

**DOI:** 10.1007/s00216-025-05951-8

**Published:** 2025-06-11

**Authors:** Jorge A. Custodio-Mendoza, Alexandra Rangel Silva, Patryk Pokorski, Havva Aktaş, Marcin A. Kurek

**Affiliations:** 1 Institute of Agroecology and Food (IAA), Food and Health Omics, Universidade de Vigo – Campus Auga, As Lagoas s/n, 32004 Ourense, Spain; 2 Department of Technique and Food Development, Institute of Human Nutrition Sciences, Warsaw University of Life Sciences (WULS-SGGW), 02-776 Warsaw, Poland; 3 REQUIMTE/LAQV – Departamento de Química e Bioquímica, Faculdade de Ciências da Universidade do Porto, 4169-007 Porto, Portugal

**Keywords:** Anthocyanins, Bioactive compounds, Food analysis, PLE, Purple corn, UAE

## Abstract

This study presents an analytical comparison of two extraction techniques—pressurized liquid extraction (PLE) and ultrasound-assisted extraction (UAE)—for the recovery of anthocyanins from purple corn using sustainable solvents (*o*-phosphoric acid, ethanol, and water). Twelve anthocyanins were identified using high-performance liquid chromatography with ultraviolet detection and tandem mass spectrometry, confirming method specificity and selectivity. Both extraction methods were optimized using multivariate experimental designs and validated following U.S. Food and Drug Administration guidelines for analytical methods in food matrices. The protocols demonstrated excellent linearity (coefficient of determination ≥ 0.9992), low detection limits (0.30–1.70 mg/kg), and high precision and accuracy (relative standard deviation ≤ 5.4%, recoveries between 97.1 and 101.9%) at 50, 100, and 150 mg/kg. To assess their environmental and operational performance, both methods were evaluated using two quantitative tools: AGREEprep, which measures greenness in sample preparation workflows, and the Blue Applicability Grade Index (BAGI), which evaluates practical applicability. PLE achieved a higher sample throughput and lower detection limits, while UAE minimized waste and required less energy. AGREEprep scores (0.73 for PLE, 0.76 for UAE) and BAGI scores (77.5 for PLE, 72.5 for UAE) confirmed both techniques as sustainable and viable for routine analysis. These results demonstrate that both PLE and UAE provide validated, efficient, and environmentally friendly alternatives for anthocyanin extraction from plant-based matrices. The study contributes to the advancement of green sample preparation and highlights the use of structured assessment tools in optimizing analytical workflows for bioactive compound determination in food analysis.

**Graphical abstract:**

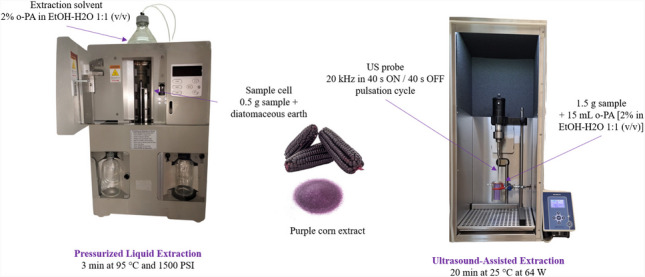

**Supplementary Information:**

The online version contains supplementary material available at 10.1007/s00216-025-05951-8.

## Introduction

Green analytical chemistry (GAC) has become increasingly relevant in response to growing environmental concerns and the demand for more sustainable scientific practices [1–3]. Founded on 12 principles, including waste minimization, safer solvents, and energy efficiency, GAC promotes environmentally responsible methodologies across all stages of chemical analysis [1, 2]. A key challenge in implementing green analytical methods lies in the sample preparation step, which is often the most resource-intensive and polluting stage of the analytical workflow [3]. To address this, green sample preparation (GSP) has emerged as a guiding framework, integrating principles such as miniaturization, automation, use of renewable and reusable materials, and in situ processing [3]. These strategies not only reduce the consumption of solvents and reagents but also lower energy requirements and improve operator safety. By minimizing ecological impact while maintaining sensitivity, selectivity, and precision, GSP contributes decisively to improving food safety and supporting human health [1–3]. In the context of food analysis, adopting greener extraction techniques for the recovery of bioactive compounds—such as anthocyanins—enhances both environmental sustainability and analytical efficiency [1, 2].

Anthocyanins, a group of water-soluble pigments found in many fruits, vegetables, and grains, are responsible for their vibrant red, purple, blue, and orange colors [4, 5]. Due to their natural origin and potential health benefits, anthocyanins have become increasingly important in health, industry, and research [4–9]. As concerns over the harmful effects of synthetic dyes grow, anthocyanins are viewed as a safer, natural alternative for food coloring [10]. Beyond their coloring properties, these compounds possess antioxidant, anti-inflammatory, and anticancer effects, which contribute to preventing and managing diseases such as cardiovascular conditions, obesity, and cancer [4, 6]. The potential health benefits of these compounds make their extraction for use in products such as juices, jams, and supplements highly relevant for both consumer health and sustainable industrial practices.

Purple corn (*Zea mays* L.), native to the Andean region, is notable for its high anthocyanin content, particularly cyanidin-3-O-glucoside (Cy3G), making it a valuable source of natural colorants and antioxidants for the food industry [5–9]. However, extracting anthocyanins from purple corn poses significant challenges [7, 11]. These compounds are sensitive to pH, temperature, light, and oxygen, which can lead to degradation during extraction [7]. Additionally, the complex composition of purple corn, which includes proteins, fibers, and other phenolic compounds, complicates the separation of anthocyanins, affecting both yield and purity [7, 11].

Furthermore, ensuring cost-effective and scalable extraction methods remains a significant hurdle for industrial applications [12]. These challenges underscore the complexity and importance of developing sustainable extraction methods for anthocyanins from purple corn that are not only efficient but also suitable for industrial use. Traditional anthocyanin extraction methods, such as maceration, rely on large amounts of organic solvents like methanol (MeOH) or acetone, often acidified with hydrochloric acid [7, 8, 10, 13, 14]. These methods are effective but involve high energy consumption and long extraction times [7, 8, 10, 13]. Moreover, they pose environmental risks due to hazardous chemicals, solvent waste, and energy-intensive processes [2, 3, 13, 14]. Sustainable extraction techniques, in contrast, aim to reduce environmental impact by using less harmful solvents, minimizing energy consumption, and increasing efficiency through miniaturization and automation [1–3, 13, 14]. These greener approaches align with GAC principles, promoting safer and more eco-friendly extraction practices [2, 3]. Techniques such as pressurized liquid extraction (PLE), ultrasound-assisted extraction (UAE), microwave-assisted extraction (MAE), and supercritical fluid extraction (SFE) have emerged as effective alternatives to conventional methods [1, 5, 9, 15, 16]. These techniques use green solvents like water, ethanol (EtOH), and CO₂, which are environmentally friendly and non-toxic [6, 9, 16]. For instance, SFE employs super-critical CO₂, often with EtOH as a co-solvent, for selective and efficient extraction with minimal environmental impact [9]. UAE uses high-frequency sound waves (cavitation) to disrupt plant cell walls, releasing anthocyanins with less solvent and shorter extraction times [16]. PLE employs high pressure and temperature to enhance solvent penetration into plant cells, leading to faster and more efficient extractions while reducing solvent usage [6].

Although PLE and UAE are well-established techniques, their optimized application with food-grade, eco-friendly solvent systems for anthocyanin extraction from purple corn remains underexplored. Several studies have examined the use of acidified water or EtOH for anthocyanin extraction in other plant matrices [5, 6, 9, 15, 16], yet comparative assessments of PLE and UAE specifically applied to purple corn—supported by full analytical validation and quantitative sustainability metrics—are scarce. Moreover, many existing protocols were not developed for this matrix and often lack the analytical rigor required for reproducibility, standardization, and transferability across laboratories. At present, no method combines optimization, validation, and structured evaluation of environmental and operational performance tailored to the complexity of purple corn. Addressing this gap is critical to support high-throughput, sustainable workflows in food analysis and to meet quality control standards in industrial settings.

In this study, we developed and validated two sustainable extraction protocols based on PLE and UAE for extracting anthocyanins from purple corn using food-grade, eco-friendly solvents. Both methods were thoroughly optimized and validated according to Food and Drug Administration (FDA) guidelines, ensuring analytical robustness, reliability, and accuracy. To our knowledge, this is the first study to offer a direct comparison of PLE and UAE applied specifically to purple corn under green chemistry criteria, integrating not only full analytical validation but also quantitative assessment of environmental and practical performance using recently proposed sustainability metrics. Our work thus fills a critical methodological gap by providing reliable, transferable, and environmentally responsible protocols tailored to a challenging and high-value matrix. These results support greener workflows in food analysis and contribute to the standardization of anthocyanin extraction methods for industrial and regulatory use.

## Experimental

### Samples and materials

Purple corn powder was purchased online from Planteon (Borków, Poland) and stored in its original packaging at room temperature, protected from light before analysis. During and after analysis, it was vacuum sealed to prevent oxidation. Unless otherwise specified, all chemicals used in this study were of high purity (≥ 98%). MeOH (CAS 67–56-1) and EtOH (CAS 64–17-5) were obtained from J.T. Baker (Philipsburg, NJ, USA). Formic acid (FA, CAS 64–18-6) and acetonitrile (ACN, CAS 75–05–8) were purchased from Poch (Gliwice, Poland). O-phosphoric acid (o-PA, > 85%, CAS 7664–38-2) and anhydrous citric acid (CA, CAS 77–92-9) were sourced from Chempure (Piekary Śląskie, Poland), while acetic acid (AA, CAS 64–19-7) was acquired from Merck (Darmstadt, Germany). Milli-Q water used in the experiments was produced using a Millipore purification system in the laboratory. Analytical standards of Cyanidin (Cy) chloride (CAS 528–58-5), Cy3G chloride (CAS 7084–24-4), Pelargonidin (Pg) chloride (CAS 134–04-3), and Peonidin 3-glucoside (Pn3G) chloride (CAS 6906–39-4) were also obtained from Merck and stored in their original packaging at 4 °C, as recommended by the manufacturer. Stock solutions of each analyte (1000 µg/mL) were prepared by dissolving the appropriate amounts in MeOH. Working solutions were prepared by diluting in 2% o-PA. All solutions were stored at 4 °C and used within 1 week.

### Pressurized liquid extraction

The PLE procedure used a Dionex ASE 200 apparatus (Sunnyvale, USA) with 5 mL stainless-steel cells. Each cell was prepared by placing two cellulose filters at the bottom. The sample (0.5 g), dispersed in 1.5 g of diatomaceous earth using a mortar and pestle, was loaded into the cell. The remaining space was filled with more diatomaceous earth, and a final cellulose filter was placed on top to complete the setup. The analytes were extracted using a mixture of 2% o-PA in EtOH and water (1:1, v/v) under optimal conditions: one static extraction cycle of 3 min at 95 °C and 1500 psi. The flush volume was 3.5 mL (70% of the cell capacity), and the purge time was 90 s. The resulting purple corn extract (approximately 5 mL) was diluted (1:9) with mobile phase, and a 1-mL aliquot was filtered through a 0.22-µm syringe filter before being analyzed by High Performance Liquid Chromatography coupled to Ultraviolet detector (HPLC–UV).

### Ultrasound-assisted extraction

The UAE process used an HD 420 ultrasound sonicator with a TS103 ultrasound probe in a 15-mL glass cell model KG3 from SONOPULS (Berlin, Germany). A total of 1.5 g of purple corn was carefully weighed into a 15-mL flask, and the volume was adjusted with a 2% o-PA (in EtOH-water 1:1 v/v) solution. The sample solution was then transferred into the glass cell, and the system’s temperature was controlled with an HBC 5 water recirculation system from IKA (Staufen im Breisgau, Germany) to ensure optimal extraction conditions: 20 min at 25 °C and 64 W, with pulsation cycles of 40 s ON/OFF at 20 kHz. A 0.1-mL aliquot of the resulting purple corn extract was diluted with mobile phase to 1 mL and filtered through a 0.22-µm syringe filter before HPLC–UV analysis.

### Maceration extraction

For qualitative purposes, the anthocyanins from purple corn were extracted following the maceration method described by de Souza Mesquita et al. (2023) [17]. In summary, 0.15 g of purple corn powder was soaked in 3 mL of water containing 0.25 mol citric acid per liter of solvent for 24 h. A 0.1-mL aliquot of the resulting extract was diluted to 1 mL with the mobile phase and filtered through a 0.22-µm syringe filter before HPLC–MS analysis.

### High-performance liquid chromatography—UV determination

Chromatographic separation was conducted following the method described by de Souza Mesquita et al. (2023) [17], with modifications. A Thermo Fisher Scientific system was employed, comprising an Accela Autosampler (60,057–60020), a quaternary pump (600), and a photodiode array (PDA) detector equipped with a 5-cm LightPipe™ flow cell operating at 40 Hz. Separation of analytes was achieved using a Kinetex C18 column (2.1 × 50 mm, 2.6 µm) from Phenomenex (CA, USA), maintained at 30 °C.

The mobile phase consisted of a 9:1 mixture of 2% o-PA in water and EtOH, operating in isocratic mode at a flow rate of 0.2 mL/min. A sample injection volume of 10 µL was used, with a total analysis time of 20 min. The PDA detector scanned wavelengths from 190 to 600 nm to capture the full UV spectrum of the analytes, with specific detection at 280 nm and 520 nm.

### Mass spectrometry for anthocyanin identification

The anthocyanins identified using the previously described method were further analyzed by mass spectrometry. This analysis was performed by coupling the UV detector to a Thermo Fisher Scientific LCQ Fleet™ Ion Trap LC/MSⁿ system. An electrospray ionization (ESI) source operated in positive ion mode was used, with the capillary temperature set to 350 °C and capillary voltage of 38 V. Nitrogen was used as sheath (21 arbitrary TSQ units), auxiliary (10 arbitrary TSQ units), and sweep (10 arbitrary TSQ units) gases. The spray voltage was 3.5 kV, and high-purity helium (≥ 99.99%) was employed as the collision gas.

Collision-induced dissociation (CID) was carried out in MS^2^ mode with a normalized collision energy of 35%, an isolation width of 2.0 m*/z*, and an activation time of 30 ms. Full-scan MS and MS^2^ spectra were acquired in SCAN mode over the *m/z* range of 50–1000. For each anthocyanin, product ion spectra were collected based on the precursor ions identified in MS mode, using data-dependent acquisition. Instrument control and data analysis were performed using Xcalibur software (Thermo Fisher Scientific). Identification of anthocyanins was supported by comparison with analytical standards (when available) and published spectral data.

### Analytical validation

Analytical figures of merit were assessed for four anthocyanins determined in purple corn extract for which there are commercially available analytical standards: Cy, Cy3G, Pg, and Pn3G. Method validation was carried out using diluted (1:10 v/v) purple corn extract as a matrix blank, which was fortified with analytical standards at designated levels to assess each parameter. The analytical method validation was performed following FDA guidelines for the validation of chemical methods in food, feed, cosmetics, and veterinary products, as well as the Bioanalytical Method Validation Guidance for Industry [18, 19]. The key figures of merit assessed included method specificity, limits of detection (LOD), limits of quantification (LOQ), linearity, accuracy, and precision. Method specificity was evaluated by selecting the maximum UV wavelength for each analyte, ensuring that the analytes eluted at distinct retention times during chromatographic separation. This separation confirmed the absence of interference from other substances, demonstrating the method’s ability to distinguish and quantify each analyte in the complex matrix. The LOD and the lower limits of quantification (LLOQ) were determined through matrix-matched calibration curves of at least six different concentration levels for each analyte within the linear dynamic range (1–200 mg/kg), which were constructed from the LLOQ to the upper limit of quantification (ULOQ) for each analyte. The LOD and LLOQ were estimated as 3.3 and 10 times the blank response’s standard deviation plus the blank response’s mean, respectively. Method accuracy was determined by using quality control samples (QCs) at three concentration levels: 50 mg/kg (QC1), 100 mg/kg (QC2), and 150 mg/kg (QC3). The accuracy was evaluated by calculating the recovery of each analyte based on quintuplicate measurements (*n* = 5). Precision was assessed through intraday and interday assays, with results expressed as the percent relative standard deviation (%RSD) based on five replicates (*n* = 5). The %RSD values confirmed that the methodologies maintained consistency and repeatability over time.

### Experimental design, statistical analysis, and performance metrics

All statistical analyses were carried out using Microsoft Excel and NemrodW® statistical software [20]. Method optimization was performed using purple corn extract spiked with a mix of analytical standards at 100 mg/kg. A preliminary factor-by-factor optimization was conducted to define the approximate working ranges of key variables for each extraction technique. This step helped establish baseline conditions from which a more efficient multivariate optimization could proceed. For the PLE method, a Doehlert experimental design was employed to optimize extraction conditions by evaluating the effects of time and temperature—two key variables identified through preliminary univariate analysis. Total anthocyanin content was used as the response variable to assess extraction efficiency. Optimal conditions were determined using a multi-criteria decision analysis based on desirability functions [20], which allowed for the simultaneous optimization of multiple goals, including maximizing anthocyanin yield while minimizing solvent consumption and extraction time. For the UAE method, an asymmetrical 3^4^4^1^ screening design was employed to investigate the effects of five variables: ultrasound amplitude, extraction time, temperature, sample size, and pulsation. A total of 12 experiments were sufficient to identify the most influential variables for further optimization. Effectiveness was evaluated based on the extraction yield and quality of anthocyanins.

AGREEprep tool, an open-access software designed to evaluate the sustainability of methods, was used to assess the environmental impact of the sample preparation method [21]. This assessment involves ten criteria, each scored between 0 and 1 (poorest to best performance). With default weightings for each criterion, these scores are combined into an overall score (0–1), where 1 indicates optimal environmental performance [22]. This tool helped identify areas for improvement in making the sample preparation more eco-friendly. The Blue Applicability Grade Index (BAGI), accessible through an online app (https://bagi-index.anvil.app/), was used to evaluate the practical applicability of the analytical methods. BAGI assesses ten critical factors divided into two main categories: analytical determination and sample preparation. Each factor is scored on a color-coded scale from 0 to 100, with dark blue indicating high applicability (10 points) and white representing low applicability (2.5 points). This tool provided a comprehensive evaluation of the methods’ feasibility, helping to pinpoint strengths and areas for improvement in the practicality of the procedures [23].

## Results and discussion

### Anthocyanin identification of the purple corn extract

**Fig. 1:**
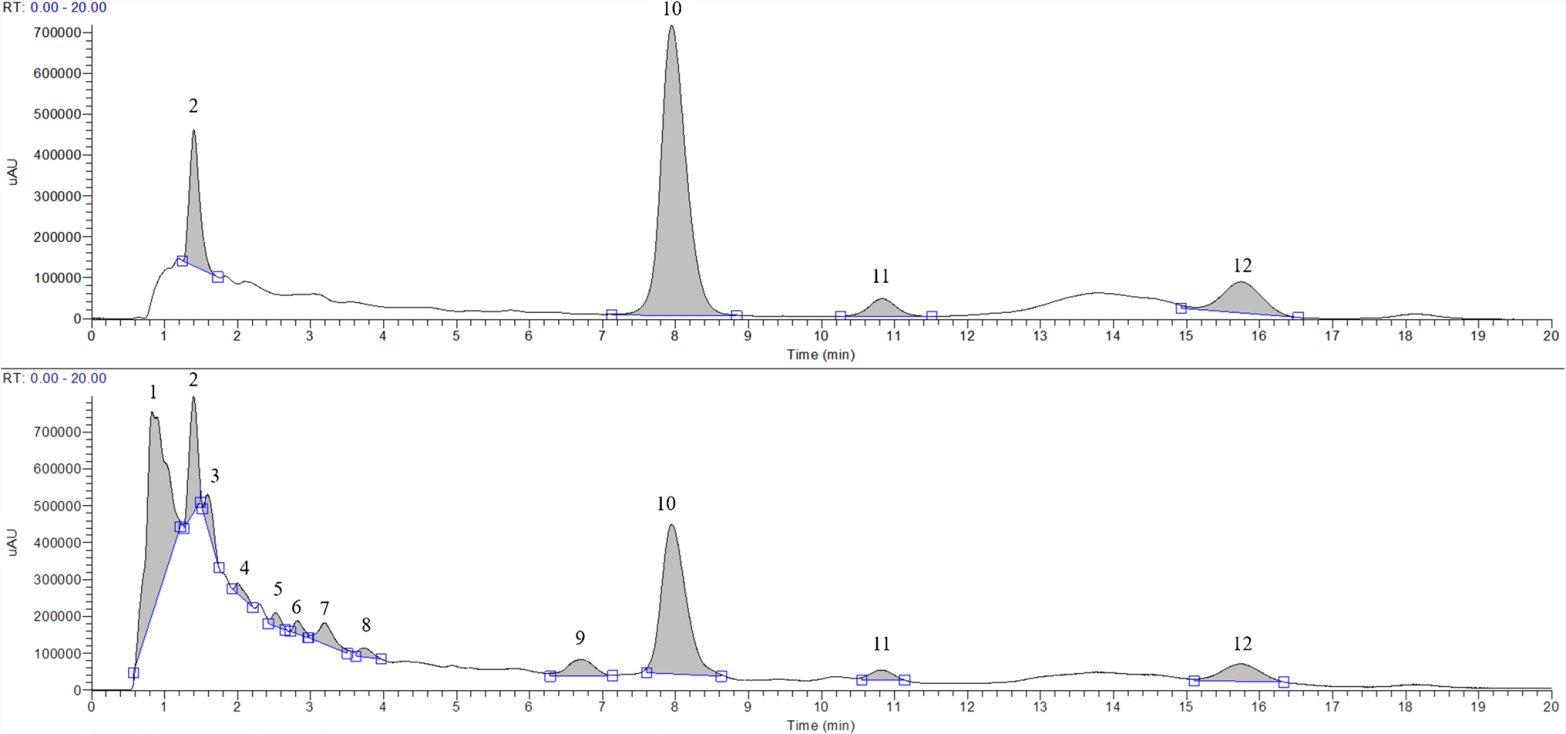
HPLC–UV chromatograms showing the maximum absorption spectra of the standard mix (10 µg/mL, top) and a purple corn extract obtained by maceration (bottom). (1) Catechin-(4,8)-cyanidin-3,5-diglucoside, (2) Cyanidin-3-O-glucoside, (3) Pelargonidin-3-O-glucoside, (4) Cyanidin-3-O-(6-malonylglucoside), (5) Pelargonidin-3-O-(6-malonylglucoside), (6) Cyanidin-3-O-(3,6-dimalonylglucoside), (7) Catechin-(4,8)-cyanidin-3,5-diglucoside, (8) Cyanidin-3-succinylglucoside, (9) Peonidin-3-(6-malonylglucoside), (10) Peonidin-3-O-glucoside, (11) cyanidin, and (12) pelargonidin

The anthocyanin profile of purple corn was determined using UV and MS spectra (Table S1, Fig. 1).

Both non-acylated and acylated anthocyanins were identified in the purple corn extract, in accordance with previous reports [5, 10, 16, 24] and based on the following observations: Peak 1 exhibited UV absorption maxima at 527 nm and 279 nm, characteristic of anthocyanins in the visible (500–540 nm) and ultraviolet (260–290 nm) regions [25]. MS/MS analysis revealed a precursor ion at 899 m*/z* and fragment ions at 287.0 m*/z*, 737.2 m*/z*, and 575.1 m*/z*. The ion at 287.0 m*/z* corresponds to cyanidin, formed by the loss of a 612 Da molecular ion. This profile is consistent with Catechin-(4,8)-cyanidin-3,5-diglucoside (10). Peak 2 showed UV absorption maxima at 523 nm and 279 nm, with no evidence of acylation 25. The precursor ion at 449.1 m*/z* fragmented into 287.3 m*/z*, 162.1 m*/z*, and 285.1 m*/z*. The ion at 287.3 m*/z* corresponds to cyanidin, confirming the compound as cyanidin-3-O-glucoside. This identification was validated by comparison with analytical standards and is consistent with previous studies [10, 16, 24]. Peak 3 exhibited UV absorption maxima at 502 nm and 278 nm, with no acylation absorption. MS/MS analysis identified a precursor ion at 433.2 m*/z* and fragment ions at 271.0 m*/z*, 162.2 m*/z*, and 269.1 m*/z*. The ion at 271.0 m*/z* corresponds to pelargonidin, consistent with pelargonidin-3-O-glucoside 10,16. Peak 4 displayed UV absorption maxima at 517 nm and 292 nm, along with an acylation-to-visible absorbance ratio (E_acyl_/E_vis_) ratio of 11%, indicating monoacylation at the sixth position [26]. MS/MS analysis showed a precursor ion at 535.0 m*/z* and fragment ions at 287.2 m*/z*, 373.2 m*/z*, and 449.2 m*/z*. This profile corresponds to cyanidin-3-O-(6-malonylglucoside) [5, 10, 16, 24]. Peak 5 had UV absorption maxima at 502 nm and 292 nm, with an E_acyl_/E_vis_ ratio of 12%, also indicative of monoacylation [26]. The precursor ion was identified at 519.1 m*/z* with fragment ions at 271.0 m*/z*, 433.0 m*/z*, and 357.1 m*/z*, corresponding to pelargonidin-3-O-(6-malonylglucoside) [5, 10, 16, 24]. Peak 6 exhibited UV absorption maxima at 517 nm and 298 nm, with an E_acyl_/E_vis_ ratio of 13%, indicating monoacylation [26]. The precursor ion was 621.2 m*/z*, with fragment ions at 287.1 m*/z*, 535.1 m*/z*, and 449.0 m*/z*, consistent with cyanidin-3-O-(3,6-dimalonylglucoside) [16, 24]. Peak 7 was identified as Catechin-(4,8)-cyanidin-3,5-diglucoside. This peak had a precursor ion at 899.1 m*/z* and fragment ions at 287.0 m*/z*, 737.0 m*/z*, and 575.1 m*/z* [10]. The UV absorption maxima were observed at 507 nm and 280 nm, with no evidence of acylation. Peak 8 corresponded to cyanidin-3-succinylglucoside, with a precursor ion at 549.2 m*/z* and fragment ions at 387.2 m*/z* and 262.1 m*/z*. Two UV absorption maximums were observed at 513 nm and 277 nm, with no acylation absorption [10]. Peak 9 showed acylation absorption at 292 nm and an E_acyl_/E_vis_ ratio of 12%. The precursor ion at 549.1 m*/z* fragmented into ions at 463.2 m*/z*, 387.1 m*/z*, and 301.0 m*/z*, consistent with peonidin-3-(6-malonylglucoside) [5, 24, 26]. Peak 10 showed UV absorption maxima at 517 nm and 282 nm, with no acylation absorption. This peak was identified as peonidin-3-O-glucoside. The precursor ion was 463.1 m*/z*, with fragment ions at 301.1 m*/z* and 192.0 m*/z*. This identification was confirmed by comparison with analytical standards [5, 10, 16, 24]. Peaks 11 and 12 exhibited UV–Vis maxima at 272 nm and 275 nm, respectively, with no acylation absorption. Peak 11 had a molecular ion of 287.1 m*/z* with fragment ions at 213.1 m*/z*, 232.2 m*/z*, and 259.1 m*/z*, corresponding to cyanidin. Peak 12 had a molecular ion of 271.1 m*/z* with fragment ions at 243.0 m*/z*, 216.2 m*/z*, and 197.2 m*/z*, corresponding to pelargonidin [10]. Both identifications were confirmed by analytical standards. Other minor signals were observed, but their intensity was insufficient for reliable identification.

A summary of the anthocyanin identification, including spectral data and confidence levels, is provided in Table S1 (Supplementary materials). Following the classification system proposed by Schymanski et al. (2014), 12 compounds were identified and assigned to two confidence levels. Four compounds were identified as Level 1 – Confirmed Structure, when commercial standards were available and confirmation was achieved through matching MS/MS spectra and retention time. While the remaining eight compounds were assigned to Level 2 – Probable Structure, when identification was based on UV–Vis absorbance patterns, MS/MS fragmentation, and comparison with literature data specific to purple corn anthocyanins [27]. It should be noted that the presence of isomeric anthocyanins with identical MS and UV–Vis characteristics represents a limitation of the current analytical approach, as full chromatographic resolution of all isomers was not achieved. This challenge is well recognized in anthocyanin analysis and may require advanced two-dimensional LC techniques to overcome [28].

### Optimization of the extraction procedures

The main objective of this research was to establish optimal extraction conditions with a reduced environmental impact compared to conventional methods. While optimal parameters vary depending on the extraction technique, certain factors—such as the type and concentration of acid and organic solvent used—play a critical role in minimizing the environmental footprint of anthocyanin extraction [1, 3]. This study evaluated four alternatives to the commonly used HCl (1–10%): two inorganic acids, CA and o-PA, and two weak organic acids, FA and AA. CA, FA, and AA are classified as green solvents due to their biodegradability, non-toxicity, and natural origin, making them more environmentally friendly than synthetic or highly corrosive acids [29]. Although o-PA is not formally categorized as a green solvent, it is recognized as Generally Recognized as Safe (GRAS) by the FDA for use in food products, and thus it is a safe option within regulated limits [30]. All acids were tested in triplicate at a concentration of 5% v/v (Fig. S1A). Similar relative chromatographic areas were observed for Cy3G, CA, and o-PA; however, o-PA yielded the highest signals for other anthocyanins, making it the acid of choice for subsequent studies.

Three levels of o-PA concentration were tested, 2%, 5%, and 10%, showing comparable responses in terms of relative chromatographic area (Fig. S1B). This consistency is attributed to the stable pH across tested concentrations, driven by the proximity to the first pKa (2.15) of o-PA. Given the similar performance at all levels, the lowest concentration (2%) was selected to reduce reagent consumption. Although most anthocyanin extraction protocols use acidified MeOH, EtOH is sometimes used due to its status as a greener solvent. Both solvents produced similar relative chromatographic areas (Fig. S1C), leading to the selection of EtOH for further experiments. Additionally, organic-to-aqueous solvent ratios of 2:1, 1:1, and 1:2 v/v were tested (Fig. S1D). Higher organic content generally yielded better responses; however, with 33% organic solvent, relative chromatographic areas remained below 80%, whereas 50% organic solvent resulted in values exceeding 80% for all analytes. Therefore, a 1:1 ratio was selected as a compromise between extraction efficiency and environmental sustainability.

#### Optimization of the PLE procedure

Optimization of the PLE procedure began with a stepwise, factor-by-factor approach focusing on four critical parameters: sample size, cell volume, clean-up sorbent composition, and number of extraction cycles (Fig. S2). As shown in Fig. S2A, reducing the sample from 1 g to 0.5 g yielded similar relative chromatographic areas, while 0.25 g resulted in peak areas below 60%. Thus, 0.5 g was selected to reduce sample usage without compromising performance. Regarding cell volume, both 5 mL and 10 mL cells were evaluated at the same mass-to-volume ratio. As results were comparable, the 5 mL cell was chosen to minimize solvent use (Fig. S2B), enhancing sustainability. For extraction cycles, Fig. S2C shows that two offered no efficiency gain over one; therefore, a single cycle was selected to reduce time and resource use.

**Fig. 2:**
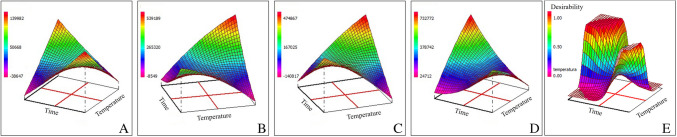
Optimization of pressurized liquid extraction conditions for anthocyanin recovery from purple corn using response surface methodology. **A** Cyanidin-3-O-glucoside, **B** Peonidin-3-O-glucoside, **C** cyanidin, **D** pelargonidin, **E** global desirability

Time and temperature, two critical parameters in anthocyanin extraction via PLE, were further optimized using surface response methodology (SRM). This approach evaluated the interaction between temperature (25–95 °C) and extraction time (1–9 min) using a Doehlert design over eight experiments. As shown in Fig. 2A–D, the response surfaces exhibit saddle points, indicating regions where the response surface transitions between local maxima and minima, reflecting complex factor interactions. These saddle points suggest that small changes in temperature or time can produce opposing effects, particularly in the extraction of Cy3G, which reached optimal recovery at both high temperature/short time and low temperature/long time combinations. To integrate the individual response surfaces into a unified optimization framework, a desirability function approach was applied. The desirability function aggregates the responses of each target compound into a single, composite response, allowing for simultaneous optimization of multiple analytes. The global desirability plot (Fig. 2E) was used to identify the optimal extraction conditions by maximizing the combined response of all target anthocyanins, thus providing a comprehensive assessment of method performance across multiple compounds. To prevent thermal degradation of anthocyanins—known to occur around 100 °C with prolonged exposure [7]—and to improve throughput by enabling more samples per hour, the final optimal extraction conditions were set at 95 °C for 3 min. This setting balanced extraction efficiency with environmental impact.

#### Optimization of the UAE procedure

**Fig. 3:**
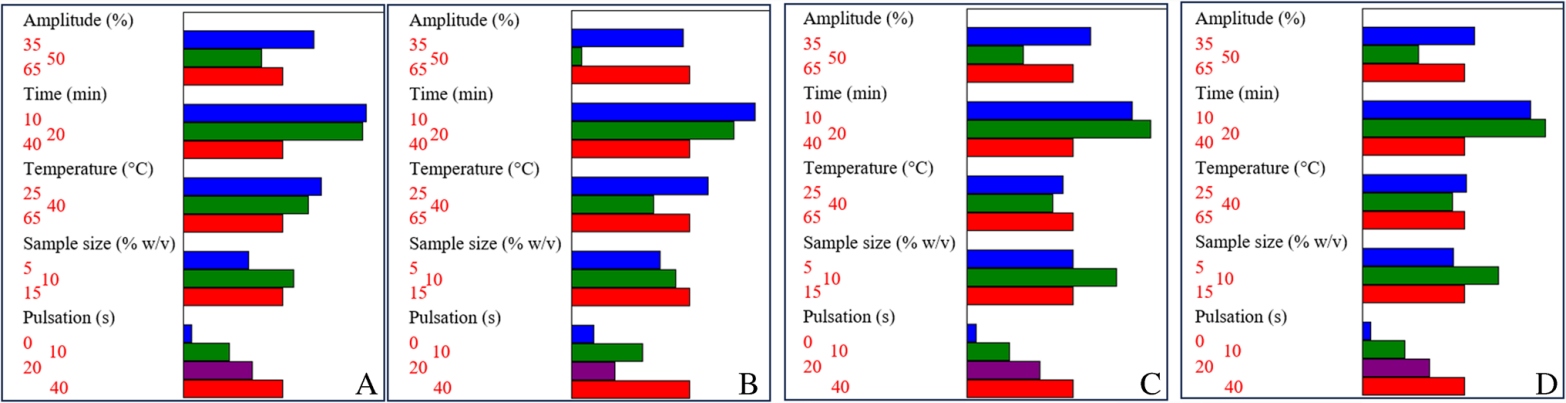
Total effect plots from asymmetrical 3^4^4^1^//12 screening design in the optimization of ultrasound-assisted anthocyanin extraction from purple corn. **A** Cyanidin-3-glucoside, **B** Peonidin-3-O-glucoside, **C** cyanidin, **D** pelargonidin

Parameters affecting the efficiency of the UAE method were studied using an asymmetrical 3^4^4^1^ screening design across 12 experiments. The variables included ultrasound amplitude (35%, 50%, 65%), extraction time (10, 20, 40 min), temperature (25 °C, 40 °C, 65 °C), sample concentration (5%, 10%, 15% w/v), and pulsation (0, 10, 20, 40 s). Although delta weight plots (Fig. S3) indicated that none of these factors had statistically significant effects on extraction efficiency, Pareto charts from the screening design revealed trends that identified the most favorable conditions (Fig. 3).

The optimal conditions were as follows: 20-min extraction at 25 °C, using a 10% w/v sample concentration, 35% amplitude (resulting in ultrasound power of 64 W), and a pulsation cycle of 40 s on/40 s off. The use of an ultrasound probe provided precise control over specific parameters such as energy input (amplitude) and the activation-deactivation cycles of ultrasonic stirring—features that are not achievable with standard ultrasonic baths. Moreover, external temperature regulation helped minimize fluctuations caused by cavitation, which could otherwise lead to anthocyanin degradation during extraction.

### Analytical figures of merit

The specific retention times and UV maxima for each anthocyanin, as shown in Table S1, confirm the method’s specificity and selectivity. Limits of detection, linearity, accuracy, and precision for both methods are detailed in Table 1.

**Table 1:** Determination Limits and linearity of the pressurized liquid extraction and the ultrasound-assisted extraction of anthocyanins from purple corn

Analyte	Pressurized liquid extraction						Ultrasound-accelerated extraction					
	LOD	LLOQ	ULOQ	m	b	r^2^	LOD	LLOQ	ULOQ	m	b	r^2^
	mg/kg	mg/kg	mg/kg				mg/kg	mg/kg	mg/kg			
Cy3G	0.40	0.97	200	1926.3	1359.2	0.9998	1.33	3.01	200	1724.1	1728.5	0.9997
Pn3G	0.30	0.98	200	2955.9	3978.7	0.9992	1.34	2.84	200	2420.5	1814.9	0.9998
Cy	1.60	5.00	200	6624.9	5733.3	0.9995	2.58	8.80	200	6332.1	7253.5	0.9994
Pg	1.70	5.20	200	7375.6	1816.9	0.9995	2.38	7.10	200	5715.0	4905.2	0.9995

Matrix-match calibration; *m*, slope; *b*, intercept; *r*^*2*^, coefficient of determination; *Cy3G*, Cyanidin 3-O-glucoside; *Pn3G*, Peonidin 3-glucoside; *Cy*, cyanidin; *Pg*, pelargonidin

Due to the presence of endogenous anthocyanins in purple corn, a completely blank matrix could not be obtained. Therefore, a matrix-matched calibration approach was applied using the diluted purple corn extract as the matrix blank. The calibration curve was constructed by fortifying this matrix at, at least, six levels of concentration starting from LLOQ: 1, 5, 10, 50, 100, 150, 200 mg/kg. The LOD and LLOQ calculations accounted for the background signal, as the standard deviation and mean values were derived directly from replicate measurements of the level 0 (unfortified extract). The PLE method achieved a slightly lower LLOQ than UAE, demonstrating excellent determination coefficients (r^2^ ≥ 0.9992) within the linear range from LLOQ to 200 mg/kg. Quality controls (QCs) were used to assess accuracy and precision (Table 2), with recovery rates ranging from 97.1% to 101.8% for the PLE method and 98.7% to 101.9% for the UAE method, all within acceptable criteria. Intraday and inter-day precision showed excellent %RSD values, with PLE showing slightly better intraday precision. Although matrix-matched calibration was used to compensate for any matrix effects that might impact anthocyanin quantification, we compared the slopes of calibration curves prepared in pure solvent (without matrix) with those of matrix-matched calibration curves to further assess potential matrix effects. The results (Table 2) indicate a slight matrix effect for Cy in both methods, confirming the need for matrix-matched calibration in each case. Previous studies on anthocyanin extraction from various foods (Table S3) have reported analytical performance metrics. However, the lack of commercially available analytical standards for all anthocyanins limits these studies, as most rely on the same standards used here, regardless of sample composition. Some studies have reported higher instrumental detection limits than those presented here [17, 31, 32], while others have reported sample detection limits that are similar to or higher than ours [33–37].

**Table 2:** Precision, accuracy, and matrix effect of the pressurized liquid extraction and the ultrasound-assisted extraction of anthocyanins from purple corn

	Analyte	Intraday precision (*n* = 5) %RSD			Interday precision (*n* = 5) %RSD			Accuracy (*n* = 5) %Recovery			Matrix effect* %
		QC1	QC2	QC3	QC1	QC2	QC3	QC1	QC2	QC3	
PLE	Cy3G	3.4	1.0	0.9	2.6	0.7	1.9	100.5	101.2	99.3	88.8
	Pn3G	1.7	2.3	2.5	3.5	1.8	3.2	102.8	101.1	98.9	89.4
	Cy	3.2	2.7	2.1	2.3	0.6	5.4	97.1	99.2	100.7	75.8
	Pg	3.0	1.4	2.4	1.6	1.4	2.4	99.4	101.8	98.9	90.2
UAE	Cy3G	2.2	1.7	1.6	2.3	0.6	5.4	99.9	101.9	99.0	86.2
	Pn3G	3.9	1.9	2.0	3.1	2.3	1.0	98.5	101.4	99.3	85.4
	Cy	3.5	3.5	2.0	4.6	2.1	2.4	101.9	99.6	99.7	58.7
	Pg	2.2	1.9	2.2	3.3	1.5	5.1	100.8	101.5	98.7	83.3

*RSD*, relative standard deviation; *QC1*, 50 mg/kg; *QC2*, 100 mg/kg; *QC3*, 150 mg/kg; *Cy3G*, Cyanidin 3-O-glucoside; *Pn3G*, Peonidin 3-glucoside; *Cy*, cyanidin; *Pg*, pelargonidin

*Calculated as a comparison of the slope of the matrix match calibration of each analyte with the external standard calibration

The methods proposed here are more accurate and precise than most previous reports, with recovery rates closer to 100% and %RSD ≤ 5.1%, attributed to the higher automation offered by PLE and UAE. Additionally, the use of UHPLC contributes to improved accuracy and precision as previously demonstrated [17, 34].

### Sustainability and viability evaluation of PLE and UAE procedures

**Fig. 4:**
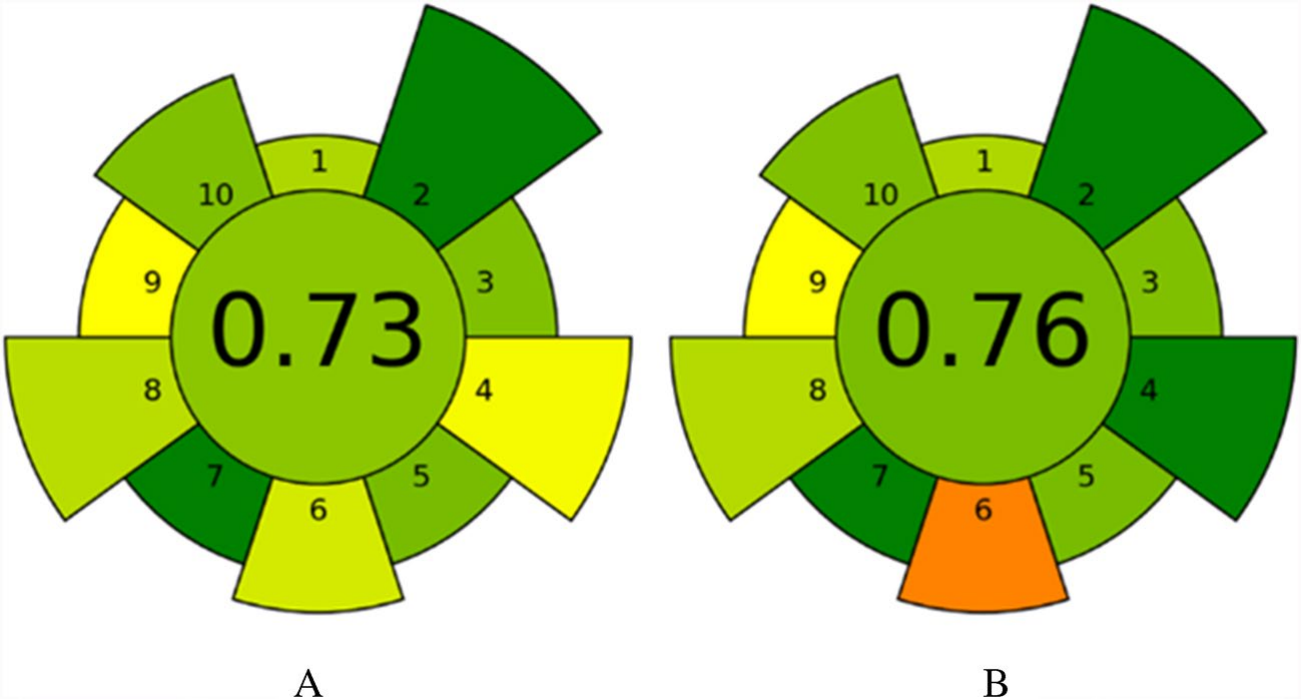
Assessment of the environmental impact of the PLE (**A**) and UAE (**B**) sample preparation methods using the AGREEprep software. The evaluation includes ten green chemistry criteria: (1) favor in situ sample preparation; (2) use of safer solvents and reagents; (3) target sustainable, reusable, and renewable materials; (4) minimize waste; (5) minimize sample, chemical, and material amounts; (6) maximize sample throughput; (7) integrate steps and promote automation; (8) minimize energy consumption; (9) choose the greenest possible post-sample preparation configuration for analysis; and (10) ensure safe procedures for the operator. Scores range from 0 (least sustainable) to 1 (most sustainable), with higher overall scores reflecting superior environmental performance

The AGREEprep assessment, performed using the open-access AGREEprep software, was employed to evaluate the environmental impact of our sample preparation methods. This tool identifies strengths and weaknesses across a series of green chemistry criteria, promoting the development of more eco-friendly sample preparation processes [21, 22]. Figure 4 presents the AGREEprep results for the environmental assessment of the PLE (A) and UAE (B) procedures in anthocyanin extraction from purple corn.

The PLE-HPLC–UV method achieved an overall score of 0.73, reflecting its strong performance in key areas such as the absence of hazardous materials (score: 1.0) and the use of a single-step, fully automated process (score: 1.0). Similarly, the UAE-HPLC–UV method scored 0.76, with strengths including the absence of hazardous materials (score: 1.0) and zero waste generation (score: 1.0). While both methods achieved comparable scores due to their use of environmentally benign reagents and simplified protocols, notable distinctions emerged in their sustainability profiles. The UAE method demonstrated higher sustainability in waste management (factor 4) due to its non-waste-generating nature, whereas the PLE method, although generating minimal waste, requires filling materials in its extraction cells. However, PLE stood out for its lower energy consumption per sample per hour, making it a more energy-efficient alternative. Both methods present environmentally superior options for anthocyanin extraction from purple corn compared to traditional approaches.

**Fig. 5:**
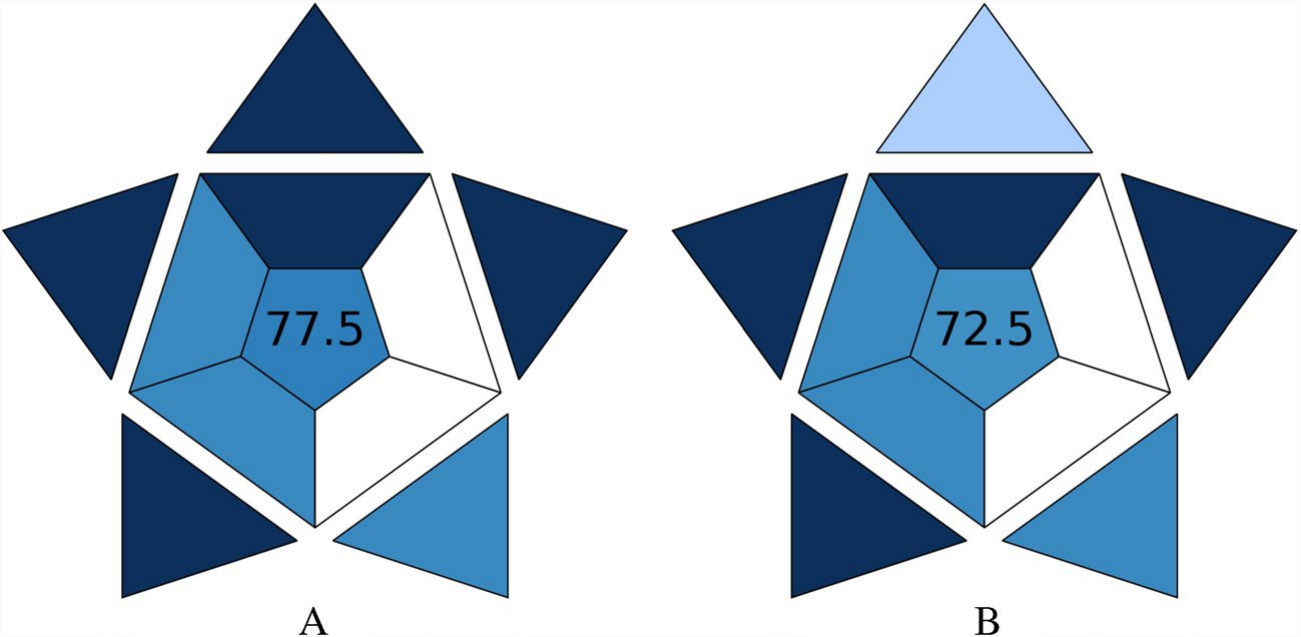
Evaluation of the practical applicability of the PLE (**A**) and UAE (**B**) methods using the BAGI online tool. BAGI assesses ten critical criteria: (1) the type of analysis, (2) the number of analytes simultaneously determined, (3) the analytical technique and required instrumentation, (4) the number of samples that can be simultaneously treated, (5) the sample preparation process, (6) the number of samples that can be analyzed per hour, (7) the type of reagents and materials used, (8) the requirement for pre-concentration, (9) the degree of automation, and (10) the amount of sample. Each attribute is color-coded on a scale from 0 to 100, where dark blue indicates high applicability (score: 10) and white indicates low applicability (score: 2.5)

In addition to environmental assessment, the practical applicability of the analytical methods was evaluated using the BAGI online app [23]. BAGI evaluates ten key attributes grouped into two main categories: analytical determination (e.g., type of analysis, number of analytes, required instrumentation) and sample preparation (e.g., sample handling capacity, reagents used, degree of automation). Figure 5 illustrates the BAGI results for the PLE (A) and UAE (B) procedures.

The PLE-HPLC–UV method obtained an overall score of 77.5, while the UAE-HPLC–UV method scored 72.5. These values reflect several practical advantages common to both techniques, including simultaneous confirmatory and quantitative analysis (factor 1, score 10), use of sustainable reagents (factor 7, score 10), no requirement for pre-concentration steps (factor 8, score 10), and the use of small sample volumes (less than 10 mL of food material, factor 10, score 10). In addition, the PLE method distinguished itself by processing a greater number of samples per hour than UAE (factor 6, score 10).

Table S3 presents a comparative overview of various analytical methods used for anthocyanin extraction and determination from purple corn. The reported methods vary significantly in terms of sample size, solvent systems, clean-up steps, and instrumentation, highlighting the diversity of approaches and their respective limitations. Maceration remains the most frequently employed technique, as evidenced by multiple studies using hydrochloric acid or acidified ethanol as extraction solvents [7, 8, 10, 36], despite growing interest in more sustainable techniques such as MAE, PLE, and SFE [5, 6, 9, 15, 16, 38]. While effective, these methods often require large sample amounts (up to 50 g) and extensive clean-up steps, such as LLE or SPE, increasing solvent consumption and environmental burden [5–10, 15, 16]. In contrast, our PLE and UAE protocols are designed to reduce sample size to 0.5 g while employing food-grade solvents (ethanol/water) without additional clean-up steps, aligning with green chemistry principles. It should be noted that SFE offers a promising alternative by utilizing CO₂ as a solvent, often combined with ethanol and water as co-solvents, minimizing the use of organic solvents [9]. However, SFE systems require higher initial costs and specialized equipment, limiting their accessibility for routine analyses. In contrast, our PLE and UAE methods use widely available laboratory equipment and standard analytical instrumentation, making them more feasible for implementation in routine workflows. On the other hand, MAE has been reported to achieve high extraction efficiencies for anthocyanins in purple corn [15, 16, 38], yet it typically involves concentrated acid solutions (0.1–0.22 M HCl) that can contribute to analyte degradation and pose handling hazards [16, 36]. In comparison, our methods employ food-grade acids recognized as GRAS and utilize milder extraction conditions, mitigating potential degradation and promoting sample integrity.

Additionally, while several studies employ mass spectrometry for detection [5, 8, 15, 16], our work demonstrates that comparable precision and accuracy can be achieved using HPLC–UV, reducing instrumentation costs while maintaining analytical performance.

## Conclusions

This study developed and validated two green sample preparation methods—pressurized liquid extraction (PLE) and ultrasound-assisted extraction (UAE)—for the extraction of anthocyanins from purple corn using food-grade solvents and HPLC–UV detection. Twelve anthocyanins were identified and confirmed via UV and tandem mass spectrometry, demonstrating the specificity and robustness of both protocols. Method optimization was guided by multivariable strategies: surface response methodology for PLE and an asymmetrical screening design for UAE. Both approaches achieved a balance between analytical performance and environmental sustainability.

Analytical validation followed U.S. FDA guidelines, confirming excellent linearity, precision, and accuracy (recoveries: 97.1–101.9%). PLE offered lower detection limits and slightly superior repeatability, while UAE minimized energy consumption and waste. AGREEprep and BAGI tools were employed not as adjunct evaluations, but as integral components of a structured framework for assessing greenness and operational feasibility. Their results (AGREEprep scores: 0.73–0.76; BAGI scores: 72.5–77.5) confirm both methods as practical, sustainable, and transferable to routine analytical workflows.

Unlike many food science-oriented studies, this work applies rigorous analytical validation and introduces standardized, sustainability-assessed workflows suitable for regulatory monitoring and industrial quality control. The dual evaluation of method performance and environmental impact demonstrates how analytical chemistry can contribute proactively to green innovation. Particularly, PLE shows strong potential for high-throughput and occurrence studies. Although the developed methodologies were applied to a single, commercially available purple corn powder, which was used for method optimization, validation, and comparison between PLE and UAE, further validation across different batches or alternative red/purple food matrices would strengthen their general applicability after the corresponding matrix-extension study. Overall, this study advances the development of reproducible, scalable, and eco-friendly analytical methods for bioactive compound determination in complex plant matrices.

## Supplementary Information

Below is the link to the electronic supplementary material.

ESM 1 (DOCX 174 KB)

## Data Availability

Data are available from the corresponding author on request.
