# Supplementary material for: Green sample preparation for anthocyanin extraction from purple corn: analytical evaluation of pressurized liquid and ultrasound-assisted extraction using sustainable solvents
Source: Anal Bioanal Chem. 2025 Jun 11;417(18):4025–37. doi: 10.1007/s00216-025-05951-8 (PMC12276115; doi:10.1007/s00216-025-05951-8)
Supplement: Supplementary file 1 — (DOCX 174 KB) [file 216_2025_5951_MOESM1_ESM.docx]

**Green sample preparation for anthocyanin extraction from purple corn: analytical evaluation of pressurized liquid and ultrasound‑assisted extraction using sustainable solvents**

Jorge A. Custodio-Mendoza^1,2*^, Alexandra Rangel Silva^3^, Patryk Pokorski^2^, Havva Aktaş^2^, and Marcin A. Kurek^2*^

^1^ Institute of Agroecology and Food (IAA), Food and Health Omics, Universidade de Vigo – Campus Auga, As Lagoas s/n, 32004, Ourense, Spain

^2^ Department of Technique and Food Development, Institute of Human Nutrition Sciences, Warsaw University of Life Sciences (WULS-SGGW), 02-776 Warsaw, Poland

^3^ REQUIMTE/LAQV – Departamento de Química e Bioquímica, Faculdade de Ciências da Universidade do Porto, 4169-007, Portugal

* Correspondence to: [jorgeantonio.custodio@uvigo.gal](mailto:jorgeantonio.custodio@uvigo.gal) (JACM); [marcin_kurek@sggw.edu.pl](mailto:marcin_kurek@sggw.edu.pl) (MAK)

**Supplementary Material**

**Table S1. Identification parameter of the Purple corn’s Anthocyanin Content.**

| **Peak** | **Compound name** | **Molecular formula** | **Retention time** | **Max UV** | **PI** | **Fragment ions** | | | **Confidence Level*** |
| --- | --- | --- | --- | --- | --- | --- | --- | --- | --- |
|  |  |  | **min** | **nm** | **m/z** | **m/z** | **m/z** | **m/z** |  |
| 1 | Catechin-(4,8)-cyanidin-3,5-diglucoside | C_39_H_41_O_21_ | 0.90 | 527/279 | 899.1 | 287.0 | 737.2 | 575.1 | 2 |
| 2 | Cyanidin-3-glucoside | C_21_H_21_O_11_ | 1.43 | 523/279 | 449.1 | 287.3 | 162.1 | 285.1 | 1 |
| 3 | Pelargonidin-3-O-glucoside | C_21_H_21_O_10_ | 1.60 | 502/278 | 433.2 | 271.0 | 269.1 | 162.2 | 2 |
| 4 | Cyanidin-3-O- (6-malonylglucoside) | C_24_H_23_O_14_ | 2.04 | 517/292 | 535.0 | 287.2 | 373.2 | 449.2 | 2 |
| 5 | Pelargonidin-3-O-(6-malonylglucoside) | C_24_H_23_O_13_ | 2.36 | 502/298 | 519.1 | 271.0 | 433.0 | 357.1 | 2 |
| 6 | Cyanidin-3-O-(3,6-dimalonylglucoside) | C_27_H_25_O_16_ | 2.58 | 517/298 | 621.2 | 287.1 | 535.1 | 449.0 | 2 |
| 7 | Catechin-(4,8)-cyanidin-3,5-diglucoside | C_39_H_31_O_18_ | 2.89 | 507/280 | 899.1 | 287.0 | 737.0 | 575.1 | 2 |
| 8 | Cyanidin-3-succinylglucoside | C_24_H_25_O_13_ | 3.82 | 513/277 | 549.2 | 387.2 | 262.1 | 100.0 | 2 |
| 9 | peonidin-3-(6-malonylglucoside) | C_25_H_25_O_13_ | 3.27 | 517/292 | 549.1 | 463.2 | 387.1 | 301.0 | 2 |
| 10 | Peonidin-3-O-glucoside | C_22_H_23_O_11_ | 8.31 | 517/282 | 463.1 | 301.1 | 192.0 | 283.1 | 1 |
| 11 | Cyanidin | C_15_H_11_O_6_ | 11.28 | 272 | 287.1 | 213.1 | 232.2 | 259.1 | 1 |
| 12 | Pelargonidin | C_15_H_11_O_5_ | 16.47 | 275 | 271.1 | 243.0 | 216.2 | 197.2 | 1 |

* Following the classification system proposed by Schymanski et al. (2014), the identified compounds were assigned to two confidence levels: Level 1 – Confirmed Structure, when commercial standards were available and confirmation was achieved through matching on UV–Vis absorbance patterns, MS/MS spectra and retention time; and Level 2 – Probable Structure, when identification was based on UV–Vis absorbance patterns, MS/MS fragmentation, and comparison with literature data specific to purple corn anthocyanins.

**Figure S1.** Factor-by-Factor optimization of the general conditions for anthocyanin extraction from purple corn using green solvents. A. type of acid studied, B. acid concentration, C. organic solvent, and D. Organic-to-aqueous ratio. CA, citric acid; o-PA, o-phosphoric acid; FA, formic acid; AA, acetic acid; MeOH, methanol; EtOH, ethanol. Each factor was assessed by triplicate and the sample spiked at 100 mg/Kg. Cyanidin 3-O-glucoside (Cy3G), Pelargonidin 3,5-di-O-glucoside (Pg3,5GG), Peonidin 3-glucoside (Pn3C), Cyanidin (Cy).

Cy3G Pg3,5GG Pn3C Cy

**Figure S2.** Preliminary Factor-by-Factor optimization of the Pressurize Liquid Extraction of for anthocyanin from purple corn using green solvents. A. Sample size, B. Cell volume, and C. Cycles of extraction. Each factor was assessed by triplicate and the sample spiked at 100 mg/Kg. Cyanidin 3-O-glucoside (Cy3G), Pelargonidin 3,5-di-O-glucoside (Pg3,5GG), Peonidin 3-glucoside (Pn3C), Cyanidin (Cy).


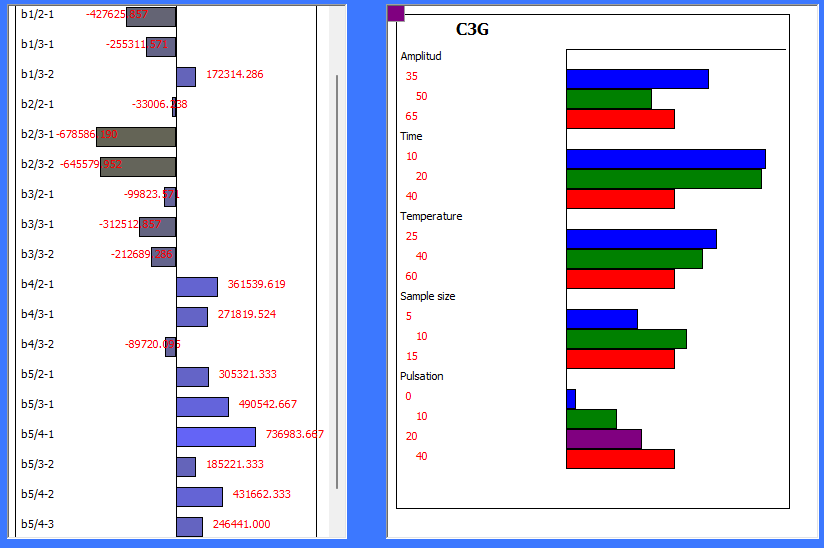

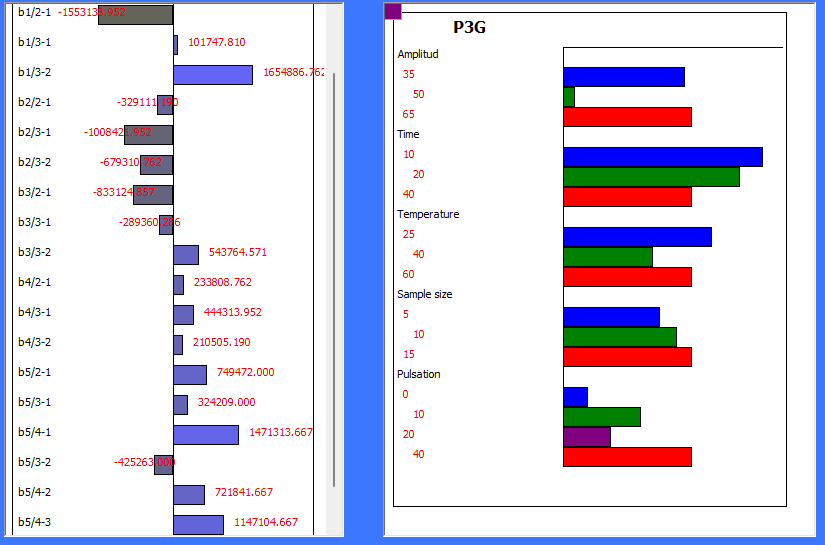

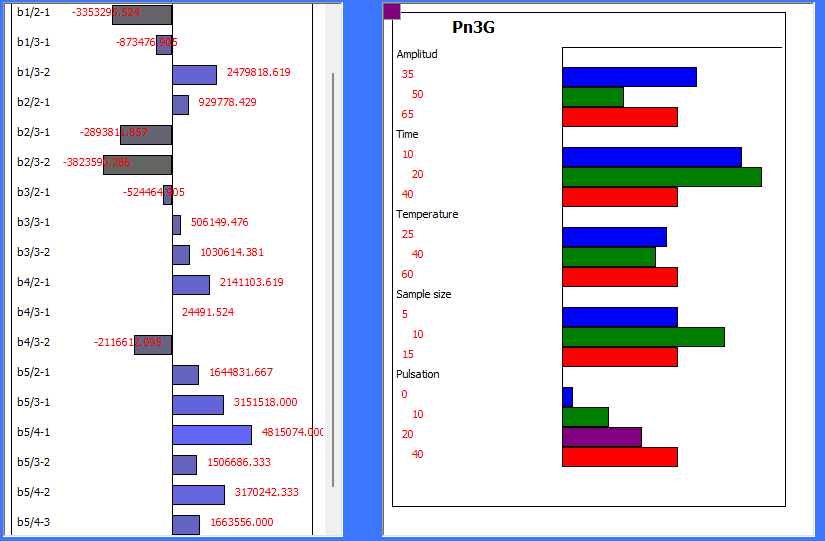

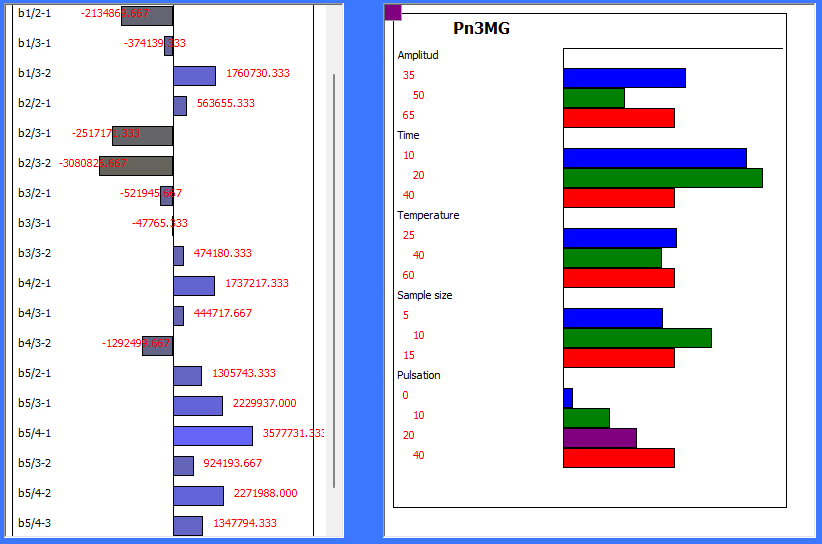


**Figure S3**. Delta weight Plots from Asymmetrical 3^4^4^1^//12 Screening Design in the Optimization of Ultrasound-Assisted Anthocyanin Extraction from Purple Corn

**Table S2. Comparison of the analytical featured for anthocyanins quantification in food.**

| **Sample** | **Anthocyanin** | **LOQ**  **ng/mL** | **r^2^** | **Precision**  **%RSD** | **Accuracy**  **%Recovery** | **Reference** |
| --- | --- | --- | --- | --- | --- | --- |
| Various food | Cy3G | 14.5* | 0.9996 | 0.53-0.61 | 98.06 | 17 |
| Sweetcorn | Cy3G  Pn3G | 150*  90* | >0.999 | 1.4-3.7  1.7-4.9 | 93-104  96-102 | 29 |
| Pomegranate | Cy3G  Pg | 67*  88* | 0.9999 | 0.1-1.3  0.2-1.4 | 96-100  99-100 | 30 |
| Strawberry | Cy3G  Cy  Pg | 4590  2590  250 | >0.998 | 2.6-6.0  0.9-6.3  1.5-7.6 | 83-85  NR  NR | 31 |
| Berries | Cy3G | 360 | 0.9999 | 0.5-1.7 | 100-101 | 32 |
| *Neomitranthes obscura* fruits | Cy3G | 70 | 0.9998 | 1.0-84 | 94 | 33 |
| Blueberry | Cy  Pg | 50  120 | > 0.9996 | 1.3-2.5  1.7-2.8 | 91-107 | 34 |
| Euterpe edulis | Cy3G | 14.8 | 0.9958 | 3.57 | NR | 35 |
| Purple corn | Cy3G  Pn3G  Cy  Pg | 10*  10*  50*  50* | >0.9997  >0.9995  >0.9992  >0.9994 | 0.6-3.4  1.0-3.9  0.6-4.6  1.4-5.1 | 99-101  98-102  97-101  98-101 | This work |

Cy3G, cyanidin-3-O-Glucoside; Pn3G; Peonidin, 3-O-Glucoside; cy, cyanidin. *Instrumental limit; NR, not reported

**Table S3. Comparison of analytical methods for anthocyanins determination in purple corn.**

| **Extraction method** | **Sample size** | **Solvent** | **Clean-up** | **Instrument** | **Reference** |
| --- | --- | --- | --- | --- | --- |
| UAE | 5g | 74% EtOH | Ammonium sulfate precipitation and ultrafiltration | HPLC-MS | 6 |
| Maceration | 1g | 0.06M HCl in EtOH (50%) | LLE | HPLC-UV | 7 |
| Maceration | 2g | 0.07M HCl in EtOH (15%) | Filtration | HPLC-HRMS | 8 |
| SFE | 5 g | CO2/EtOH/water 68:22:10 %v/v/v | Non | HPLC-UV | 9 |
| Maceration | 50 g | 0.06M HCl in acetone (70%) | LLE with hexane +SPE | HPLC-UV-MS | 10 |
| PLE and MAE | 1 g | 80% Acetone | Non | HPLC-UV-MS | 15 |
| UAE | 1g | 0.1M citric acid in EtOH (95%) | column chromatography | HPLC-UV-MS | 16 |
| MAE | 10 g | 0.22 M HCl in EtOH | Ion-exchange column chromatography | HPLC-MS | 38 |

MAE, Microwave-Assisted Extraction; PLE, Pressurized Liquid Extraction (also referred to as Accelerated Solvent Extraction, ASE); SCE, Supercritical Fluid Extraction; HPLC, High-Performance Liquid Chromatography; UV, Ultraviolet Determination; MS, Mass Spectrometry; HRMS, High-Resolution Mass Spectrometry.
